# Enhanced oral bioavailability of two Cistanche polysaccharides in acteoside: an in-depth analysis of intestinal flora, short-chain fatty acids, and pharmacokinetic regulation

**DOI:** 10.3389/fnut.2025.1509734

**Published:** 2025-05-02

**Authors:** Jing Lian, Yuan Zhang, Kexu Dong, Ji Shi, Fan Zhang, Guoshun Shan, Pengpeng Liu, Nan Wang, Tianzhu Jia

**Affiliations:** ^1^Liaoning University of Traditional Chinese Medicine, Dalian, China; ^2^Key Research Laboratory of Traditional Chinese Medicine Processing Process Principles of the State Administration of Traditional Chinese Medicine, Dalian, China; ^3^Liaoning Provincial Traditional Chinese Medicine Processing Professional Technology Innovation Center, Dalian, China

**Keywords:** *Cistanche deserticola*, acteoside, polysaccharides, pharmacokinetics study, gut microbiota

## Abstract

**Introduction:**

For centuries, *Cistanche deserticola* Y. C. Ma has been considered to have the effect of “tonifying the kidney and strengthening the yang,” and is used for the prevention and treatment of diseases such as impotence, female infertility, lumbago and senile constipation. Polysaccharides and small molecules of acteoside are the main chemical compounds co-existing in *Cistanche deserticola* Y. C. Ma with health benefits, but the interaction of these two compounds *in vivo* is not yet known.

**Methods:**

Here, we investigated the effects of unprocessed Cistanche polysaccharides and wine-processed Cistanche polysaccharides on the metabolism of acteoside *in vivo* through gut microbiota. Male Sprague–Dawley rats were randomly divided into Control group, unprocessed Cistanche polysaccharide group (UCP group) and wine-processed Cistanche polysaccharide group (WCP group). After 21 days of intervention with unprocessed Cistanche polysaccharides and wine-processed Cistanche polysaccharides, rats were given 100 mg/kg of acteoside on day 22. Acteoside and its associated metabolites pharmacokinetically studied were analysed using UPLC-QqQ-MS, and the composition of short-chain fatty acids (SCFAs) in excrement was measured using the technique of GC–MS. The microbiological composition of the intestines was discovered using 16S rRNA gene amplicon sequencing.

**Results:**

The results showed that the Cistanche polysaccharides used in this experiment, including unprocessed Cistanche polysaccharides and wine-processed Cistanche polysaccharides, could regulate the diversity of gut microbiota and increase the number of beneficial bacteria, especially wine-processed Cistanche polysaccharides were able to promote the growth of *Ligilactobacillus* and *Duncaniella* genus, and improve the production of SCFAs and the absorption of acteoside.

**Discussion:**

By exploring the synergistic effects of large molecules Cistanche polysaccharides and small molecule acteoside, this paper provides a new explanation for the scientific use of plant-derived polysaccharides to improve the bioavailability of oral drugs.

## 1 Introduction

*Cistanche deserticola* Y.C. Ma (CD), a perennial psammophytic species endemic to arid regions of China, Mongolia, Japan, and other East Asian territories, holds a distinguished position in traditional medicine systems spanning over two millennia. Revered as the principal constituent of the renowned *Wubishuyu* Pill in the classical medical compendium <*Beiji Qianjin Yaofang*>, CD demonstrates particular prominence in reproductive health formulations such as the *Suoyang Gujing Pill* - a testament to its historical application in replenishing kidney yang, enhancing essence-blood nourishment, and modulating immune resilience. Modern pharmacological investigations substantiate these traditional claims through evidence of multifaceted bioactivities including neuroprotection, immunomodulation, endocrine regulation, anti-fatigue effects, hepatoprotection, antioxidant capacity, antimicrobial/antiviral properties, and antineoplastic potential (1). Phytochemical analyses reveal acetoside, a phenylethanoid glycoside demonstrating remarkable biological versatility through its demonstrated antioxidant, anticonvulsant, neuroprotective, anti-inflammatory, antifungal, antihypertensive, and immunoenhancing properties. Cistanche polysaccharides have a variety of pharmacological activities, including immunomodulation, antioxidant, anti-aging, hepatoprotective, anti-inflammatory and intestinal microbiota regulation (2, 3). As this species’ predominant bioactive component alongside polysaccharide macromolecules.

Current pharmacopoeial standards record raw *Cistanche deserticola* and its wine-steamed product. Pharmacodynamic studies demonstrate that steam by rice wine induces significant phytochemical transformations, notably a marked reduction in acteoside content coupled with a substantial increase in isoacteoside levels (4). This compositional shift correlates with enhanced therapeutic efficacy in kidney yang-tonifying applications, suggesting potential synergistic enhancement through processing. Furthermore, structural modification of polysaccharide components during wine steaming has been demonstrated to substantially improve antioxidant capacity (5), though the molecular mechanisms underlying this phenomenon remain incompletely elucidated. Notably, the potential synergistic interaction between bioactive polysaccharides and phenylethanoid glycosides, particularly isoacteoside, represents a critically understudied area requiring systematic investigation.

Emerging evidence suggests that acteoside’s therapeutic efficacy is constrained by its limited bioavailability and rapid hepatic metabolism following oral administration, prompting investigations into complementary bioactive components capable of enhancing systemic delivery. Non-digestible polysaccharides from Traditional Chinese Medicine (TCM) have emerged as particularly promising candidates due to their unique structural characteristics that enable gastrointestinal resistance while modulating gut microbiota composition and metabolic activity (6, 7), thereby functioning as prebiotic agents that facilitate herbal compound absorption through microbial biotransformation (8). This symbiotic relationship is exemplified by multiple TCM systems where polysaccharides demonstrate synergistic interactions with bioactive small molecules: Ginseng polysaccharides enhance ginsenoside Rb1 bioavailability through microbiota-mediated metabolism, ameliorating dextran sulfate sodium (DSS)-induced colitis in rodent models (9); soybean-derived polysaccharides improve genistein bioavailability while attenuating high-fat diet-induced adiposity and metabolic dysfunction in murine studies (7); lotus root polysaccharides (LRPs) demonstrate antioxidant synergism with phenolic compounds through microbial fermentation (10); and Astragalus polysaccharides (APS) enhance flavonoid bioavailability via gastrointestinal stabilization and permeation enhancement (11). Building upon this foundational knowledge, our study specifically investigates two critical aspects of *Cistanche deserticola* Y.C. Ma (CD) polysaccharide functionality, the differential effects of raw versus wine-processed CD polysaccharides on gut microbiota profiles, and processing-induced alterations in acteoside’s metabolic pathways and bioactive transformations that may influence its pharmacokinetic behavior and therapeutic potential.

**Figure 1:**
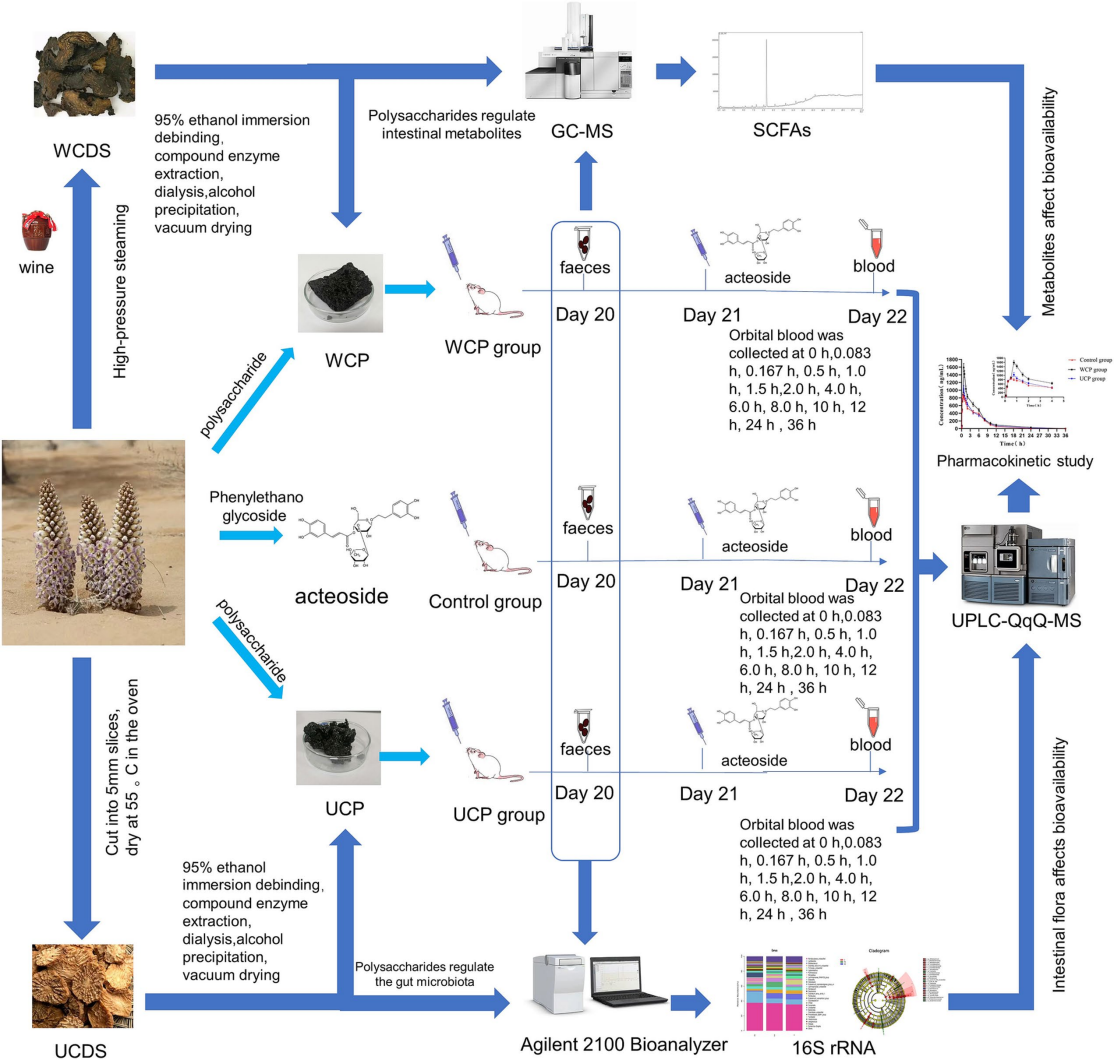
Experimental scheme. Cistanche polysaccharides regulate gut microbiota to increase acteoside bioavailability: an in-depth analysis of intestinal flora, short-chain fatty acids, and pharmacokinetic regulation.

This study systematically investigates the synergistic mechanisms between polysaccharides and acteoside in *Cistanche deserticola* through integrated multi-omics analysis. The experimental design scheme is shown in Figure 1. We developed a UPLC-QqQ-MS method for simultaneous quantification of acteoside metabolites (caffeic acid and hydroxytyrosol) in biological samples, coupled with 16S rRNA sequencing and SCFA metabolomics to profile gut microbiota modulation. Comparative evaluation of rice wine-processed versus raw polysaccharides revealed enhanced bioavailability enhancement correlated with structural characteristics of processed polysaccharides. Our findings establish a mechanistic framework for optimizing polysaccharide-phenylethanoid glycoside synergy through controlled processing, demonstrating enhanced therapeutic index potential for Cistanche-derived nutraceuticals.

## 2 Materials and methods

### 2.1 Materials

*Cistanche deserticola* Y.C. Ma was collected from Alashan mengrongshantang cistanche Co. Ltd. The samples were identified by Prof. Yanjun Zhai (Liaoning university of Traditional Chinese Medicine). The plant specimens (The herbarium voucher number of 20,210,508-111) were deposited in the herbarium of the Technical Innovation Center of Traditional Chinese Medicine Processing, Liaoning University of Traditional Chinese Medicine. China Chengdu Herb Substance Co. Ltd. was the source of the standard substances, including acteoside (NO: 220115), caffeic acid (NO: 220412), hydroxytyrosol (NO: 220214), and the internal standard genistein (NO: 220402). Fisher Scientific (Pittsburgh, PA) supplied chromatography-grade acetonitrile and methanol. Anaqua Chemical Supply Inc. (Wilmington, USA) provided the formic acid. Cellulase (batch number: C8260), Amylase (batch number: G8290), Papain (batch number: G8430) were purchased from Beijing Solebold Technology Co., Ltd. Pure water (ddH_2_O, Milli-Q^®^ IQ Element), Methyl tert-butyl ether (Lot number: 1634-04-4, HPLC grade, Shanghai Anpel Experimental Technology Co., LTD), Sulfuric acid (Lot number: 7664-93-9, Purity≥95(AR), Sinopharm Chemical Reagent Co., Ltd.) 2-Methylvaleric acid (Lot number: 97-61-0, Purity≥99.5), Acetic acid (Lot number:64-19-7, Lot number: Purity≥99.5), Propionic acid (Lot number: 79-09-4, Purity≥99.5), Isobutyric acid (Lot number: 79-31-2, Purity≥99.5), Butyric acid (Lot number: 107-92-6, Purity≥99.5), Isovaleric acid (Lot number: 503-74-2, Purity≥99.5), Valeric acid (Lot number:109-52-4, Purity≥99.5), Hexanoic acid (Lot number:142-62-1, Purity≥99.5), Heptanoicacid (Lot number: 111-14-8, Purity≥99.5), Octanoic acid (Lot number: 124-07-2, Purity≥99.5), Nonanoic acid (Lot number: 112-05-0, Purity≥99.5), Decanoic acid (Lot number: 334-48-5, Purity≥99.5) all purchased from Dr. Ehrenstorfer GmbH. Analytical grade chemical reagents comprised the remaining ones.

### 2.2 The preparation and preliminary identification of Cistanche polysaccharides

#### 2.2.1 The preparation of Cistanche polysaccharides

Unprocessed *Cistanche deserticola* slices (UCDS) and wine-processed *Cistanche deserticola* slices (WCDS) were processed according to *Chinese Pharmacopoeia* (2020 edition) (12). UCDS:Remove impurities, wash clean, moisten thoroughly, cut into 5 mm thick slices, and dry. WCDS: Take the cistanche slices and steam them according to the wine steaming method (General rule 0213) until the wine is exhausted and removed after being steamed for 4 h at 1.25 atmospheric pressure, then dried at 55°C in an oven for drying. A total of 2.0 kg of UCDS or WCDS was ground into a rough powder. Two additional times, at 60°C for 2 h each, dry coarse powder was extracted using an aqueous solution containing 95% ethanol (v/v). The proportions of solid to liquid were 1:12 and 1:10, respectively to remove fat and wax, filtered, and dried in a cool ventilated place.

The powder was immersed in purified water (material-liquid ratio 1:10) and extracted with composite enzymes (cellulase 30 g, amylase 10 g and papain 5 g) at 55°C for 1 h at reflux, and then boiling rapidly to render the enzyme inactive. A dialysis membrane (MWCO 3500 Da) was used to dialyze the permeate for 2 days after the water solution was mixed and filtered. Then concentrated under reduced pressure and then precipitated in 80% ethanol, stood under 4°C for 24 h. The precipitate was isolated through centrifugation at 4,000 rpm for 5 min. After that, the water solution was concentrated under low pressure, five times as much anhydrous ethanol was added to make the ethanol v/v fraction 80%, stood under 4°C for 24 h. Centrifugation was used to separate the precipitate for 10 min at 4,000 rpm. To obtain the unprocessed Cistanche polysaccharide (UCP), the precipitate was dried within a vacuum drier at 50°C after 3 cycles of cleaning with acetone and anhydrous ethanol. The same method is used to obtain wine-processed Cistanche polysaccharide (WCP).

#### 2.2.2 Preliminary identification of Cistanche polysaccharides

The sulfuric acid phenol method was used to determine the polysaccharide content, with glucose serving as a standard. Using the BCA method, the total protein content was calculated.


$$\text{Polysaccharide yield}\left(\%\right)=\frac{m1}{m0}\times 100\%$$

In the formula, m0 is the mass of cistanche slices before degreasing; m1 is the mass of dried crude polysaccharide.

The average molecular weight was measured by gel permeation chromatography (GPC) in combination with multiangle laser light scattering (DAWN HELEOS-II laser photometer Wyatt Technology Co., USA) and a differential refractive index detector Optilab T-rEX(RID) (Wyatt Technology Co., USA). The system was equipped with an Ohpak SB-803 + Ohpak SB-804 + Ohpak SB-805 (300 × 8 mm, 10 μm, Shodex, Japan) column. During analysis, ultrapure water was applied as the mobile phase with a flow rate of 0.3 mL/min (45°C). Data collection and analysis were performed by Wyatt Technology ARTRAV software.

The polysaccharide sample (5 mg) was dissolved in 2 M trifluoroacetic acid (TFA) and hydrolyzed in a sealed glass tube at 121°C for 2 h. The hydrolysate was dried with nitrogen, then followed by the addition of 10 mL deionized water and filtered through 0.22 μm microporous filtering film for measurement. The supernatant was injected into a ICS5000 system (Thermo Fisher, USA) with a Dionex™ CarboPac™ PA20(150*3.0 mm, 10 μm)columnn (mobile phase: A, ddH2O; B, 0.1 M NaOH; C, 0.1 M NaOH & 0.2 M NaAC; flow rate: 0.5 mL/min; column temperature:30°C) 0.13 kinds of monosaccharide standards including fucose, rhamnose, arabinose, galactose, glucose, xylose, mannose, fructose, ribose, galacturonic acid, glucuronic acid, mannuronic acid, guluronic acid were determined as references.

### 2.3 Experimental animals and sample collection

#### 2.3.1 Animals

Liaoning Changsheng Biotechnology Co. Ltd. (Laboratory Animal Resource Center of Liaoning Province), license number: SCXK 2020–0001 (Benxi, Liaoning, China) supplied male Sprague–Dawley rats (SPF grade) weighing 250 ± 20 g. Each one was raised at an animal facility with SPF housing and breeding practices. In this study, all animal experiments were conducted in accordance with ARRIVE guidelines and in accordance with the National Research Council’s Guidelines for the Care and Use of laboratory animals. The Research Ethics Committee of Liaoning University of Traditional Chinese Medicine has granted the ethical approval of animal participation in this study. (Approval No. 2020067). This study focused on the therapeutic effects of Cistanche polysaccharides and WCP on male kidney deficiency-related diseases, so male rats were selected as experimental subjects. *Cistanche deserticola* is mainly used in clinical treatment of male kidney deficiency impotence, infertility and other symptoms (13). The physiological and behavioral characteristics of male rats are relatively stable. On the one hand, female rats interfere with estrogen due to estrus cycles, the accuracy of experimental data (14); on the other hand, gender differences are the first factor affecting the intestinal barrier. The intestinal permeability of females is usually lower than that of males (15). In order to ensure the consistency and reliability of experimental results, male rats were selected in this experiment to be closer to clinical applications.

#### 2.3.2 Cistanche polysaccharide intervention and sample collection

After a week of acclimatization, rats were split into three groups at random: the wine-processed Cistanche polysaccharide group (WCP group), the unprocessed Cistanche polysaccharide group (UCP group), and the blank control group (Control group). Six rats each group were raised in the same cage for breeding. Prior to the experiment, provide water and regular laboratory meals to the rats at will and fast for one night before the experiment.

In order to ensure the continuous intake of Cistanche polysaccharides, it was calculated according to the requirements of the *Chinese Pharmacopoeia* (16) based on the daily dosage of Cistanche for adults, the polysaccharide yield of Cistanche polysaccharides and the equivalent conversion formula of the drug administration between humans and rats (17). An appropriate amount of raw Cistanche polysaccharide and Cistanche wine polysaccharide were weighed, dissolved in pure water, and prepared with 15.06 g·kg^−1^ administration solution for continuous administration for 21 days. After fasting overnight, on day 22, two fresh fecal samples were collected from each rat, put straight into a sterile tube from the anus, avoiding contact with skin or urine, and stored in a −80°C freezer immediately after freezing with liquid nitrogen for subsequent analysis. For the pharmacokinetics, in a 1.5 mL heparinized centrifuge tube, 0.3 mL of continuous blood collected from the orbital vein was taken from 0 min, 5 min, 10 min, 30 min, 1.0 h, 1.5 h, 2.0 h, 4.0 h, 6.0 h, 8.0 h, 10 h, 12 h, 24 h, and 36 h following oral administration of acteoside (100 mg/kg) in the three groups. Using a cryo-centrifuge, plasma samples were produced by centrifuging at 800 g for ten minutes at 4°C. After that, plasma samples were gathered and kept for later examination at −80°C.

### 2.4 Pharmacokinetic study

#### 2.4.1 Calibration standards and quality control (QC) sample preparation

Accurately weighed acteoside, caffeic acid, and hydroxytyrosol (1.0 mg) were put to a 1.5 mL brown centrifuge container along with 1.0 mL cold 50% methanol to dissolve and thoroughly mix. A 4°C freezer was then used to make and store the stock solution (1 mg/mL). Moreover, 50% methanol was used to dilute 1 μg/mL of the IS working solution. Acteoside, caffeic acid, and hydroxytyrosol mother liquor were successively diluted with 50% methanol to produce a mixed control series of working solutions at concentrations of 50, 250, 500, 1,000, 2,000, 5,000, 10,000 and 20,000 ng/mL. The brown centrifuge tube was filled with 5 μL of the preceding working solutions, 45 μL of blank plasma, vortexed, and combined for a minute to make standard curve samples containing acteoside, caffeic acid, and hydroxytyrosol at 5, 25, 50, 100, 200, 500, 1,000, and 2,000 ng/mL. As low, medium, and high dosage quality control samples, plasma samples with concentrations of 25, 200, and 1,000 ng/mL were employed. Before being analyzed, the entire set of QC samples were kept at −80°C.

#### 2.4.2 Preparation of plasma samples

A centrifuge was used to get 100 μL of plasma specimens for 15 min at 4°C. Following this,20 μL of genistein (1 μg/mL IS) solution and 600 μL of cold acetonitrile were then included and centrifuged for 5 min, the mutant proteins were separated by vortexing for 5 min, and then they were spun down for 15 min at 4°C at 14,000 rpm. The supernatant was gathered and dried at 40°C with a light nitrogen stream. The dried remnant was then immersed in 100 μL of 25% aqueous acetonitrile fluid, and centrifugation was performed again for 10 min at 4°C at 14,000 rpm. Ultimately, the LC–MS/MS system was injected with 2 μL of the liquid for analysis.

#### 2.4.3 UPLC-QqQ-MS analysis

A 1.7 μm and 2.1 × 100 mm I.D. ACQUITY UPLC BEH C18 column was used to separate the samples. 0.1 percent formic acid in water (solvent A) and acetonitrile (solvent B) formed the mobile phase, which had the following gradient: 0 to 4 min, 90–35% A; 4 to 5 min, 35–10% A; 5 to 6 min, 10–0% A; 6.1 to 10 min, 10% A. With a column temperature of 30°C, the flow rate was fixed at 0.3 mL/min.

**Figure 2:**
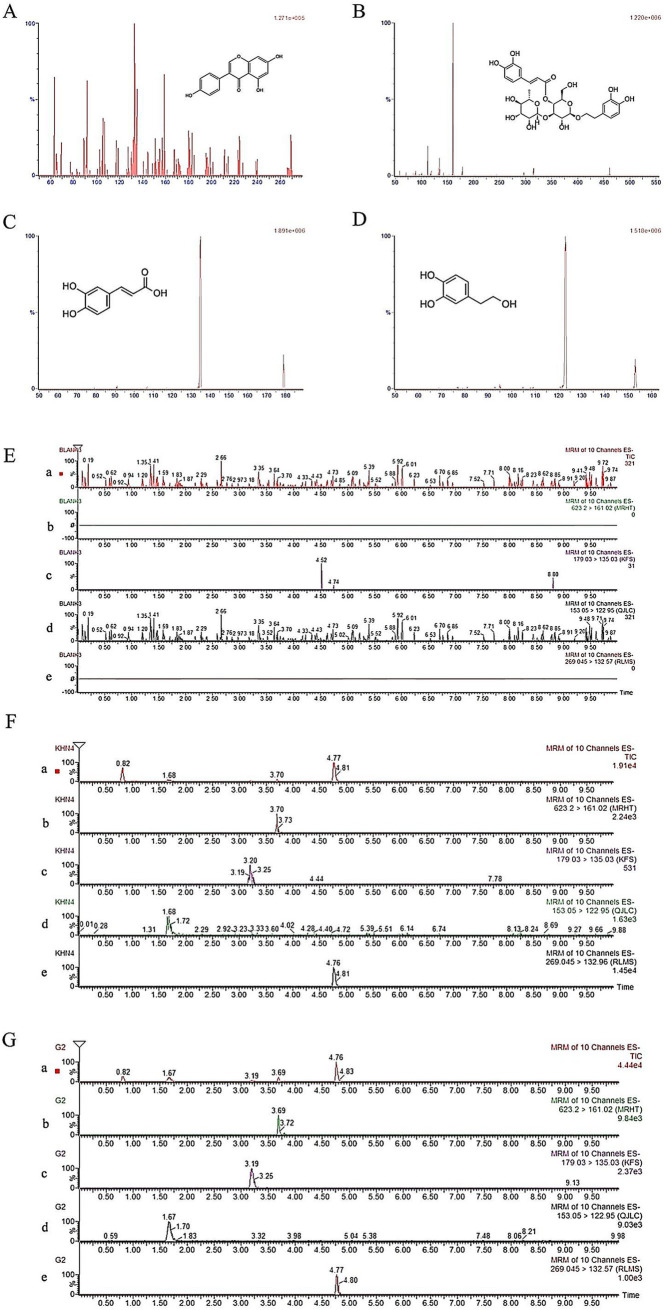
UPLC-QqQ-MS spectrums of genistein **(A)**, acteoside **(B)**, caffeic acid **(C)** and hydroxytyrosol **(D)** and instance chromatograms from blank plasma **(E)**, plasma spiking with analyte along with IS **(F)**, and plasma sample 1.0 h after acteoside oral administration **(G)** a: total ion current chromatogram; b: acteoside extract ion flow chromatogram; c: caffeic acid extract ion flow chromatogram; d: hydroxytyrosol extract ion flow chromatogram; e: genistein extract ion flow chromatogram.

In the mass spectrometry analysis, negative ionization mode was performed with the following optimized parameters: capillary voltage, 3.0 kV; desolvation temperature, 400°C. Nitrogen was used as desolvation and cone gas, at a flow rate of 800 and 30 L/h, respectively. The mass spectrometry research employed negative ionization mode, with optimum values of 400°C for the desolvation temperature and 3.0 kV for the capillary voltage. Nitrogen served as the cone gas and for desolvation, with flow rates of 30 and 800 L/h, respectively. Thus, multiple reaction monitoring (MRM) of the acteoside transitions at 623.37 → 161.02 *m/z*, caffeic acid transitions at 179.01 → 135.03 *m/z*, hydroxytyrosol transitions at 153.03 → 122.95 *m/z*, genistein transitions at 269.08 → 132.57 *m/z* was used for quantification. The cone voltage for acteoside, caffeic acid, hydroxytyrosol and genistein were 64 V, 32 V, 32 V and 56 V; 38 V, 14 V, 14 V, and 34 V were the collision energies. The mass spectra of acteoside, caffeic acid, hydroxytyrosol and genistein were shown in Figure 2. Data were processed using Masslynx workstation data acquisition and qualitative analysis software (4.1, Waters, USA).

#### 2.4.4 Validation of methods

In compliance with the International Conference on Harmonization Harmonized Guideline Bioanalytical Method Validation M10 and the U.S. Bioanalytical Method Validation Guidance for Industry, method validation was carried out to evaluate selectivity, lower limit of quantification (LLOQ), linearity, accuracy, precision, matrix effects, extraction recovery, and stability of pharmacokinetic studies.

To investigate the possible interference of endogenous chemicals with four compounds in animal plasma, the method’s selectivity was assessed. In order to accomplish this, different chromatograms were reviewed, including plasma specimens from six blank animals, blank animal plasma samples that had been mixed with an internal standard solution and control solution, and plasma samples that were taken 1.0 h after the rats had been given acteoside orally.

A concentration range of 5.00 ~ 2000.00 ng/mL was used to determine linearity. The level of the analytical substance was determined as the abscissa (X) by injecting three samples of each concentration of the standard solution. The ratio of the analytical substance’s peak area to the IS in the sample served as the ordinate (Y) to establish the standard curve. The weighted least squares linear regression method was then used to obtain the regression equation (weighted factor = 1/x^2^) By gradually diluting QC samples at low concentration levels with a signal-to-noise ratio of 3:10, the limits of detection (LOD) and quantitation (LOQ) were ascertained. A minimum limit of quantification (LLOQ) is the lowest concentration on the standard curve and the lowest amount of analyte that can be quantified with a precision (relative standard deviation, RSD) of no more than 15% and an acceptable accuracy (relative error, RE) of 85% ~ 115%.

By producing six samples in parallel at each concentration and analyzing them over the course of 1 day or three consecutive days, respectively, QC samples at low, medium, and high concentrations were used to calculate the intra-day and inter-day precision and accuracy. Precision, defined as RSD, which is, should be below 15 %, and accuracy is measured by comparing the average analyte concentration with the nominal concentration. Within 15% of the nominal value is the acceptable range for the proportion of the deviation (RE) from the average from the nominal value.

The peak areas obtained for the QC samples at low, medium, and high concentrations that were subjected to the extraction procedure were compared with those obtained from blank plasma extracts that had been spiked post-extraction at the corresponding concentrations to estimate the extraction recovery in sets of six replicates of QC samples.

An examination of stability using QC samples at low, medium, and high doses. Short-term stability was measured by injecting samples after staying 4 h at room temperature (25°C). After preparation, the sample was kept at 4°C for 24 h, and the dosage was injected to ascertain the sample’s stability. To determine long-term stability, samples were frozen at −80°C for 15 days before being injected. By thawing the samples three times in a row at room temperature and a freezer at −20°C, the freeze–thaw stability was ascertained.

#### 2.4.5 Statistical and pharmacokinetic evaluation

Using the DAS program (Chinese Pharmacological Society, Beijing, China), pharmacokinetic parameters were estimated. Every data point is displayed as mean ±SD. With SPSS 16.0, a one-way ANOVA was carried out. *p* values less than 0.05 were deemed to indicate an important variation in the data.

Statistical analysis Using the DAS program (Chinese Pharmacological Society, Beijing, China), pharmacokinetic parameters were estimated. Every data point is displayed as mean ±SD. With SPSS 16.0, a one-way ANOVA was carried out. *p* values less than 0.05 were deemed to indicate an important variation in the data. Using the DAS program (Chinese Pharmacological Society, Beijing, China), pharmacokinetic parameters were estimated. Every data point is displayed as mean ±SD. With SPSS 16.0, a one-way ANOVA was carried out. p values less than 0.05 were deemed to indicate an important variation in the data.

### 2.5 Gut microbiota structural profiling

The CTAB technique was applied to extract the DNA. After being dissolved within 50 μL of elution buffer, the whole DNA remains freezing at −80°C. Universal primers were used for sequencing and individual barcods were added to the 5′ ends of the primers for each sample. A total of 25 μL of response mixture—which included 2.5 μL of each primer, 12.5 μL of PCR Premix, and 25 ng of target DNA—was used for the PCR amplification process. The first cycle of denaturation at 98°C for 30 s, the 32 cycles of denaturation at 98°C for 10 s, cooling at 54°C for 30 s, and extension at 72°C for 45 s, and the final elongation at 72°C for 10 min were the PCR settings used for amplified the microbiological 16S sequences.2% agarose gel electrophoresis was used to validate the PCR results. To eliminate the potential for false-positive PCR results as a negative control, ultrapure water was utilized during the DNA extraction procedure in place of an experiment solution. Qubit (Invitrogen, USA) was used to quantify the PCR products after they had been purified using AMPure XT beads (Beckman Coulter Genomics, Danvers, MA, USA). Prior to sequencing, the amplicon libraries were created, and the Agilent 2,100 Bioanalyzer (Agilent, USA) and the Library Quantification Kit for Illumina (Kapa Biosciences, Woburn, MA, USA) were used to measure the amplicon library’s size and quantity. 250 bp paired-end reads were produced when the library was sequenced (Table 1) on the NovaSeq 6,000 platform.

**Table 1:** PCR primer sequences.

Region	Primers
V3-V4	341F(5′-CCTACGGGNGGCWGCAG-3′) 805R(5′-GACTACHVGGGTATCTAATCC-3′)
Archae	F(5′-GYGCASCAGKCGMGAAW-3′) R(5′-GGACTACHVGGGTWTCTAAT-3′)
V4	515F(5′-GTGYCAGCMGCCGCGGTAA-3′) 806R (5′-GGACTACHVGGGTWTCTAAT-3′)
V4-V5	F(5′-GTGCCAGCMGCCGCGG-3′) R(5′-CCGTCAATTCMTTTRAGTTT-3′)

### 2.6 Research on short-chain fatty acids (SCFAs)

Oral administration of WCP and UCP was conducted for 3 weeks in male Sprague–Dawley rats, respectively. On the twenty-second day of the trial, feces samples were gathered and promptly frozen at −80°C. Using gas chromatography (GC), the following SCFAs can be determined: acetic acid, butyric acid, decanoic acid, hexanoic acid, heptanoic acid, isovaleric acid, isobutyric acid, nonanoic acid, octanoic acid, propionic acid and valeric acid. The standardized parameters for SCFA measurements are shown in Supplementary Table S4, S5.

Enter 200 mg of feces into two milliliter EP tubes, extract with one milliliter of Milli-Q H_2_O, and vortex combine for 10 sec. Pulverized in an iron ball crusher at 40 Hz for 4 min, followed by 3 repetitions of an ultrasonic treatment for 5 min while soaking in ice. At 5000 rpm and 4°C, centrifuge for 20 min. Add 0.1 mL of 50% H_2_SO_4_ and 0.8 mL of the extraction liquid (25 mg/L stock in methyl tert-butyl ether) as an internal standard. Swirl blend for 10 s, oscillate for 10 min, and then ultrasonic treat for 10 min (incubated in cold water). Deposit the 0.8 mL of supernatant into new 2 mL EP tubes. At 4°C and 10,000 rpm, centrifuge for 15 min. Retain approximately 30 min at −20°C. For GC–MS analysis, put the supernatant into a brand-new 2 mL container. Using the SHIMADZU-GC2030-QP2020-NX GC–MS, the HP-FFAP capillary column was linked. In (5:1) fractional mode, 1 μL was the injection volume. Helium serves as the carrier gas, and its front inlet purge flow rates are 3 3 mL·min^−1^ and 1 mL·min^−1^, respectively. The predetermined heating mode was 80°C for 1 min, followed by a five-minute ramp to 200°C at 10°C/min and a one-minute ramp to 240°C at 40°C/min. The temperature of the ion source was 200°C, while the injection temperature was 240°C. Measurements from mass spectrometry were obtained in Scan/SIM mode with an electron’s impact energies of −70 eV, covering the m/z range of 30–150 Da.

### 2.7 Bioinformatics analysis

Using the Illumina NovaSeq system, specimens were sequenced in accordance with the manufacturer’s instructions. Sequence installation, data filtration, and barcodes and priming sequence cleavage in accordance with QIIME2 (18). After denoising using techniques like “DE-replication,” which is comparable to clumping with 100% similarities, DADA2 (Divisive Amplicon Denoising Algorithm) technology was employed to create an OTU (Operational Taxonomic Units) database using the idea of ASVs (Amplicon Sequence Variants). After obtaining the final ASV characteristic tables and ASV characteristic sequence, the *α*-diversity index of the intestine microbe abundance (Observed_species and Chao1 indices) and community diversity (Shannon and Simpson indicators) were calculated and evaluated using the QIIME2. Bray-Curtis lengths are employed in *β*-diversity analysis to examine modifications to structure in communities of bacteria. The Linear Discriminant Analysis Effect Size technique is intended for the identification of biomarkers and for the evaluation of sequencing data. By comparing the species abundances of various groups, the groups with notable variations were identified. The Kruskal-Wallis rank-sum test was utilized to identify all characteristic species. To determine if all subspecies of the substantially different species found in the previous phase descended to the same biological level, the Wilcoxon rank-sum test was employed. The effect size of each differential gut microbiota and the traits linked to each group of rats were determined by linear discriminant analysis (LDA), and the R package ggcorrplot was used to accomplish the ultimate differential species. Using SPSS 22.0 carried out principal coordinate analysis (PCoA) correlation analysis. An ANOVA was used to examine the SCFA data. GraphPad Prism (GraphPad Prism version 5.1) was utilized for statistical examination and graphical representation of the data. To avoid false positive results, a Kruskal-Wallis test with Bonferroni correction was employed.

## 3 Results

### 3.1 Preparation and analysis of Cistanche polysaccharides

In this study, two *Cistanche deserticola* polysaccharides were obtained by complex enzyme extraction, dialysis, alcohol precipitation, washing and drying. Table 2 displays the composition of two polysaccharides. Using a glucose standard curve, the total sugar content of the specimen was ascertained. The results showed that after being steaming by rice-wine, the yield of polysaccharides increased, the total sugar content was higher, and the molecular weight decreased significantly. *Cistanche deserticola* polysaccharides are mainly composed of arabinose, galactose, glucose, and xylose. The proportion of xylose in UCP is higher than that in WCP, while the proportion of glucose and arabinose in WCP increases.

**Table 2:** The compositions of cistanche polysaccharides.

Samples	Yield (%)	Total sugar (%)	Protein (%)	Molecular weight (kDa)	Monosaccharide constitutions (%)			
					Ara	Gal	Glc	Xyl
UCP	22.82	58.10	3.95	22.868	2.15	8.62	15.86	2.98
WCP	25.02	63.60	3.71	3.315	3.55	7.56	18.13	0.46

### 3.2 Effect of Cistanche polysaccharides on the pharmacokinetics of oral acteoside in rats

#### 3.2.1 Method validation

The representative chromatograms of blank plasma, plasma samples spiked with the analytical substance and IS, and a plasma sample taken from a rat after acteoside was administered orally in Figure 2 demonstrated the method’s selectivity by showing that there were no endogenous compound interference peaks in the chromatographic results and a good baseline resolution of the analyte acteoside, caffeic acid, hydroxytyrosol, and genistein (IS) at retention times of about 3.70 min, 3.19 min, 1.67 min, and 4.77 min, respectively.

Table 3 displayed the regression equations, linear ranges, correlation coefficients (R2), and LLOQs. Accuracy, precision, matrix effects, and extraction recoveries were shown in Table 4. In Table 3 excellent linearity across the concentration range was indicated by the R2 values for each regression equation above 0.994. The established technique for the measurement of analytes in rat plasma was found to be sensitive, as evidenced by the accuracy (RE, %) and precision (RSD, %) of the LLOQ for each analyte meeting the required standards. The analyte QC samples in Table 4 showed less than 15% intraday and interday accuracy and precision, demonstrating that the analytical approach is reliable and accurate for the pharmacokinetic investigations of analytes following oral acteoside administration. Caffeic acid, hydroxytyrosol, and acteoside extraction recoveries of QC samples ranged from 85.30 to 90.94% on average. There was no discernible matrix impact during the sample analysis, as indicated by the average matrix effect, which varied from 85.76 to 91.58%.

**Table 3:** Analytes in rat plasma: linearity and LLOQs.

Compounds	Calibration curve regression equation	R^2^	Linear range (ng/ mL)	LLOQ (ng/ mL)
Acteoside	Y1 = 0.217351X1−2.9291	0.9983	5.00~2000.00	5
Caffeic acid	Y2 = 0.425741X2+3.72924	0.9971	5.00~2000.00	5
Hydroxytyrosol	Y3 = 0.401161X3+0.176857	0.9943	5.00~2000.00	5

**Table 4:** Measurements in rat plasma: exactness, consistency, matrix influence, and extraction recovery.

Compounds	Concentration (ng/mL)	Intra-day			Inter-da			Extraction recoveries (%)	Matrix effect (%)
		Mean ± SD (ng/mL)	Precision RSD (%)	Accuracy RE (%)	Mean ± SD (ng/mL)	Precision RSD (%)	Accuracy RE (%)		
Acteoside	25	27.61 ± 1.01	3.66	10.51	28.29 ± 1.12	3.96	10.71	88.79 ± 2.55	86.98 ± 7.13
	200	203.53 ± 0.96	0.47	5.09	205.44 ± 3.59	1.75	4.15	89.33 ± 3.62	88.18 ± 8.07
	1,000	1001.24 ± 0.94	0.094	0.12	1002.31 ± 4.21	0.42	0.11	88.79 ± 2.55	85.76 ± 10.23
Caffeic acid	25	23.13 ± 1.17	5.07	10.59	22.09 ± 1.18	5.33	10.44	85.30 ± 4.32	87.42 ± 4.71
	200	198.24 ± 0.63	0.32	5.11	197.67 ± 3.85	1.95	5.35	87.65 ± 5.04	86.09 ± 6.59
	1,000	999.35 ± 1.59	0.16	0. 40	997.55 ± 4.29	0.43	0.12	87.99 ± 3.14	85.78 ± 9.13
Hydroxytyrosol	25	22.25 ± 0.85	3.84	10.77	23.17 ± 1.07	4.63	10.04	89.79 ± 6.05	89.98 ± 2.36
	200	202.54 ± 3.22	1.59	5.21	201.33 ± 4.29	2.13	6.51	90.94 ± 4.35	91.34 ± 7.03
	1,000	1001.71 ± 2.8	0.28	0.31	1002.9 ± 2.11	0.21	0.23	89.45 ± 6.55	91.58 ± 5.37

Table 5 displays the stability data, which comprise short-term, post-preparation, long-term, and freeze–thaw stability. Results reveal that the analytes in plasma samples show good stability in various circumstances.

**Table 5:** Stability of analytes under different storage conditions (n = 6).

Compounds	Concentration (ng/mL)	Short-term stability (RSD,%)	Post-preparation stability (RSD,%)	Long-term stability (RSD,%)	Freeze–thaw stability (RSD,%)
Acteoside	25	6.91	7.03	8.28	7.93
	200	3.15	3.46	6.67	4.56
	1,000	2.79	3.68	4.99	4.45
Caffeic acid	25	7.78	8.33	9.75	9.14
	200	7.34	7.87	8.56	8.23
	1,000	4.91	6.87	8.43	7.08
Hydroxytyrosol	25	5.34	6.32	7.78	7.04
	200	3.26	4.89	5.86	5.78
	1,000	2.44	3.21	4.63	4.24

#### 3.2.2 Pharmacokinetic behaviors of acteoside

Acteoside’s limited oral absorption, quick metabolism, and quick bodily excretion all lead to its incredibly low bioavailability. Acteoside’s limited oral bioavailability makes it challenging for the drug to reach an exact concentration in the blood at the point of behavior, which restricts its biological activities. The majority of glycosides are digested in the gastrointestinal system by enzymes or microbes prior to being absorbed by the body (19). In order to clarify the gavage dose of acteoside and to explore its pharmacokinetic studies, the levels of acteoside and its two metabolite compounds in rat plasma were determined rapidly and reliably by UPLC-MS. In order to achieve acceptable resolution and symmetrical peak morphologies in a comparatively short analysis time, the chromatographic conditions were first optimized by column, mobile phase, and gradient elution methods. Following multiple optimization trials, the trio of compounds achieved a precise MS identification in under 10 min of well-separation on an ACQUITY UPLC BEH C18 (2.1 × 100 mm, 1.7 μm) column using a 0.1% formic acid-acetonitrile mobile phase. Scanners operating in MRM mode with an ESI-source were used to precisely and sensitively identify the chemical structures of the acteoside and its metabolites. To achieve the highest relative abundance of parent and product ions for various analytes, the MS/MS settings and collision energies (CE) were optimized.

**Figure 3:**
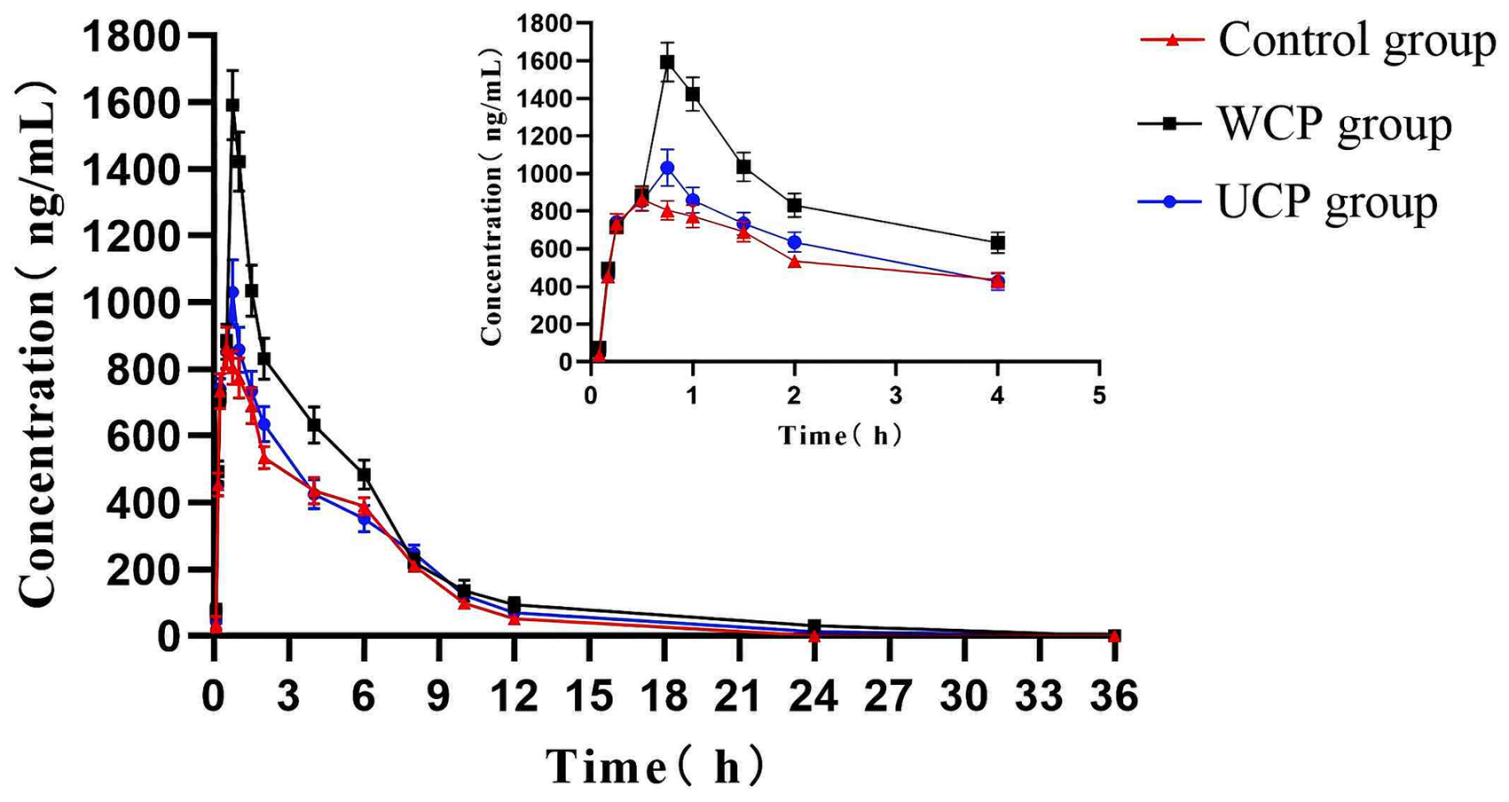
Acteoside’s mean plasma concentration-time profiles in rats following oral administration.

Rats were given acteoside orally at a dose of 100 mg/kg, and the pharmacokinetic analysis of this dosage was effectively conducted using the approved methodology. Figure 3 displayed the acteoside mean plasma concentration-time curve. The DAS system (Chinese Pharmacological Society, Beijing, China) was utilized for pharmacokinetic analysis, which involved calculating the maximum plasma concentration after administration (C_max_), drug half-life (t_1/2_), drug peak time (T_max_), and area under the plasma concentration-time curve (AUC). A two-compartment open model (weight = 1/cc) provided the best match to the primary pharmacokinetic parameters given in Table 6 based on the Akaike Information Criterion (AIC) and R^2^ values. The values of acteoside AUC (0-t), AUC (0-∞), T_max_ and C_max_ in the plasma of rats administered with Cistanche polysaccharides were significantly higher than those of contral group. The mean C_max_ of acteoside in WCP, UCP and Control groups was 1592.34 μg/L, 1031.34 μg/L and 863.45 μg/L, respectively, which was 1.84 times higher in WCP group and 1.19 times higher in UCP than control group. In addition, both T_max_ and t_1/2_ of acteoside were prolonged in the WCP and UCP groups compared to the control group. The t_1/2_ of the WCP group was 1.18 times higher than that of the UCP group.

**Table 6:** Acteoside’s mean pharmacokinetic characteristics following oral treatment (100 mg/kg) in rats (*n* = 6).

Parameters	Unit	Control	UCP	WCP
AUC(0-t)	ug/L*h	4202.886 ± 153.241	4978.446 ± 230.543	6656.577 ± 542.814
AUC(0-∞)	ug/L*h	4356.133 ± 129.766	5051.794 ± 266.616	6689.322 ± 632.723
MRT(0-t)	h	3.942 ± 0.165	4.938 ± 0.165	4.996 ± 0.032
MRT(0-∞)	h	4.337 ± 0.512	5.294 ± 0.629	5.598 ± 0.837
t_1/2_	h	2.062 ± 1.296	3. 237 ± 1.431	3. 804 ± 1.763
T_max_	h	0.50	0.75	0.75
C_max_	ug/L	863.45 ± 63.43	1031.34 ± 98.32	1592.34 ± 103.21

#### 3.2.3 Pharmacokinetic behaviors of metabolites from acteoside

**Figure 4:**
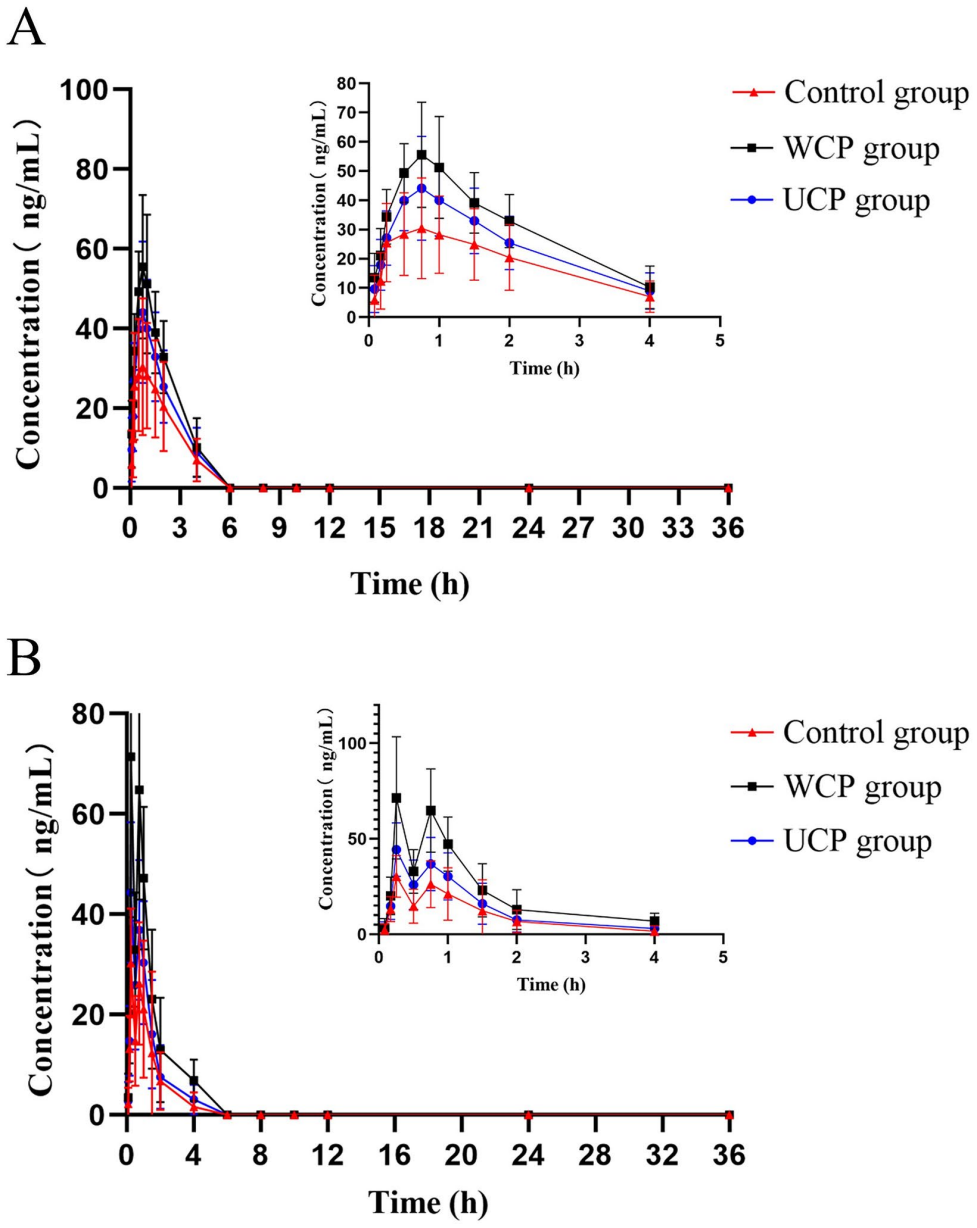
After oral delivery, mean plasma concentration-time profiles containing hydroxytyrosol **(B)** and caffeic acid **(A)** in rats.

A total of 11 metabolites, including hydroxytyrosol, hydroxytyrosol sulfate conjugation, caffeic acid, etc., have been found as a result of numerous investigations that have demonstrated the stability of acteoside in modeled gastric and intestinal liquids and its metabolism in human intestinal flora. These metabolites can be produced by eight different metabolic processes, such as deglycosylation, rhamnose removal, and hydroxytyrosol removal (20). Figure 4 displays the average plasma concentration-time curves of hydroxytyrosol and caffeic acid in the plasma of the three groups of rats following oral acteoside delivery. Figure 4A shows that, within 4 h, the WCP group’s caffeic acid content was higher than that of the UCP group and the control group. This suggests that a greater amount of acteoside in the WCP group was hydrolyzed and converted to caffeic acid through metabolism. Furthermore, as Figure 4B illustrates, within a 2-h period, the WCP and UCP groups showed higher levels of hydroxytyrosol presence than the control group. The hydroxytyrosol mean plasma concentration-time curve revealed that the pharmacokinetic curve displayed a double-peak phenomena with a brief peak time interval. The quick metabolism of acteoside in the liver following absorption-acteoside is transformed into the secondary metabolite hydroxytyrosol and subsequently released into plasma-may account for the second peak (21). Table 7 demonstrates that the UCP group’s t_1/2_ for caffeic acid was longer than that of the control group, and that the WCP and UCP groups’ AUC (0-∞) and C_max_ values for both caffeic acid and hydroxytyrosol were considerably greater than those of the control group. Compared to the UCP group, the WCP group’s C_max_ for caffeic acid was 1.26 times higher, and the C_max_ of hydroxytyrosol was 1.61 times that of the UCP group.

**Table 7:** Average pharmacokinetic characteristics of metabolites following oral acteoside delivery (100 mg/kg) in rats (*n* = 6).

Analytes	Parameters	Unit	Control	UCP	WCP
Caffeic acid	AUC(0-t)	ug/L*h	76.094 ± 13.35	99.992 ± 17.68	124.683 ± 25.89
	AUC(0-∞)	ug/L*h	109.781 ± 15.36	117.183 ± 13.35	143.676 ± 26.67
	MRT(0-t)	h	1.564 ± 0.172	1.539 ± 0.166	1.525 ± 0.161
	MRT(0-∞)	h	2.508 ± 0.273	2.182 ± 0.267	2.098 ± 0.256
	t1/2	h	2.166 ± 0.693	1.329 ± 0.824	1.289 ± 0.914
	T_max_	h	0.75	0.75	0.75
	C_max_	ug/L	30.43 ± 14.22	44.09 ± 15.75	55.54 ± 16.89
Hydroxytyrosol	AUC(0-t)	ug/L*h	40.596 ± 3.243	56.452 ± 5.413	90.562 ± 6.728
	AUC(0-∞)	ug/L*h	42.519 ± 4.243	60.485 ± 5.722	91.323 ± 6.925
	MRT(0-t)	h	1.203 ± 0.132	1.186 ± 0.124	1.266 ± 0.139
	MRT(0-∞)	h	1.383 ± 0.145	1.461 ± 0.153	1.536 ± 0.168
	t1/2	h	0.813 ± 0.129	0.908 ± 0.106	0.537 ± 0.116
	T_max_	h	0.25	0.25	0.25
	C_max_	ug/L	30.32 ± 10.92	44.36 ± 14.02	71.43 ± 32.02

### 3.3 Effect of Cistanche polysaccharides on the composition and diversity of gut microbiota

**Figure 5:**
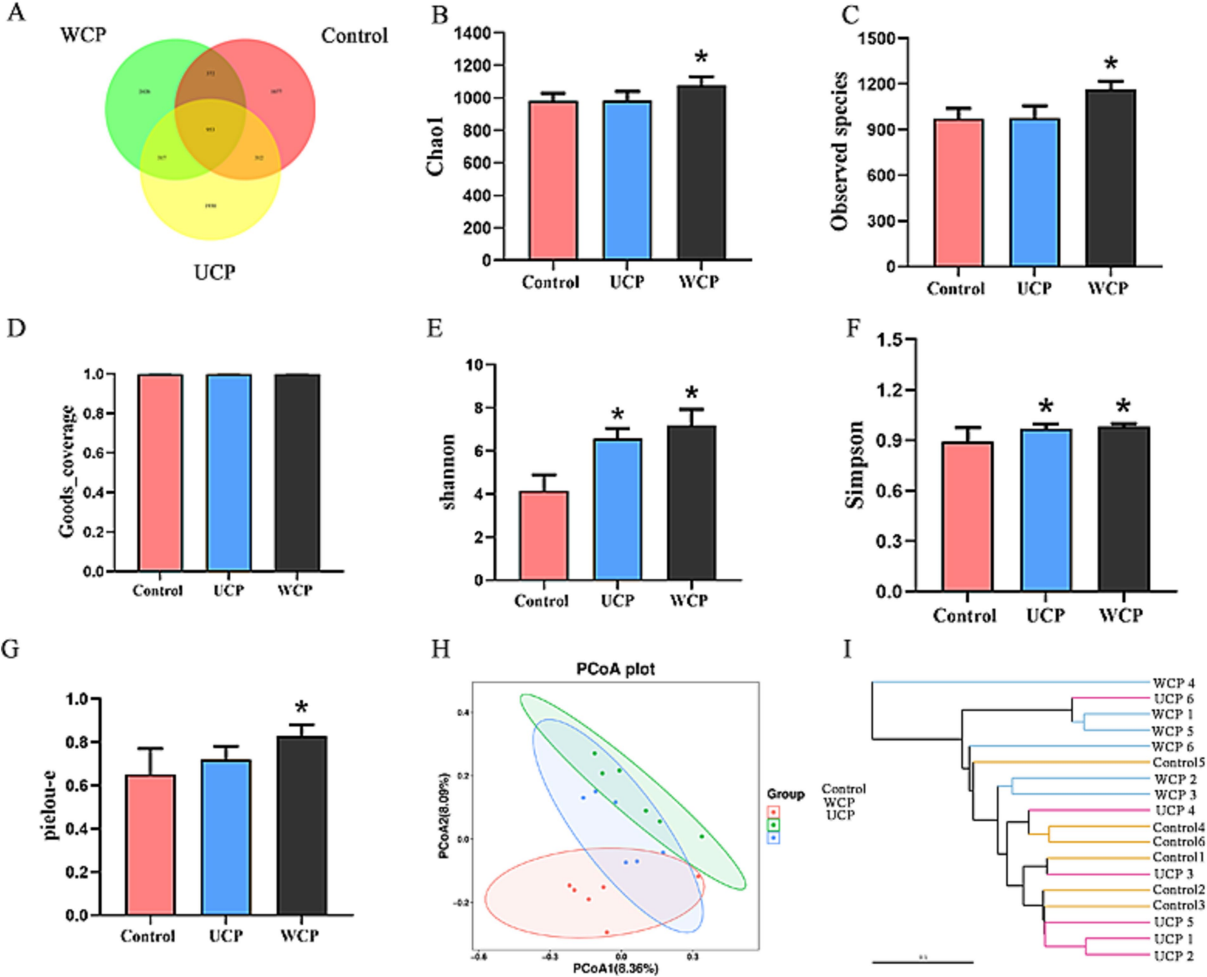
Venn diagram showing the composition of the intestinal microbiota in the WCP, UCP, and Control groups **(A)**; Effect of Cistanche polysaccharides on gut microbiota *α*-diversity: Chao1 index **(B)**, observed species index **(C)**, Goods_coverage index **(D)**, shannon index **(E)**, Simpson index **(F)** and pielou-e index **(G)**. The data is shown as mean ± SD (n = 6). Using SPSS (version 33.0) software, one-way ANOVA and the independent-samples Kruskal-Wallis test were used to identify significant differences. *p < 0.05 vs. Control group; Impact of Cistanche polysaccharides on the *β*-diversity of the gut microbiota: PCoA analysis adopting the unweighted UniFrac distances **(H)** and UPGMA applying the weighted UniFrac distance-based methods **(I)**. Six samples were represented by each plot.

Using 16S rRNA gene sequencing research to assess the impact of WCP and UCP on the microbiota in the gut. After splicing, an overall of 1.333.424 valid tags were produced; 453,507 tags in the control group, 435,105 tags in the UCP group, and 444,812 tags in the WCP group were subjected to assurance of quality and chimeric screening (Supplementary Table S1). After denoising by the DADA2 method (22), 3,148 amplicon sequence variant (ASV) numbers of ASVs in the three groups sequences were generated. The Venn diagram (Figure 5A) illustrates the different. Of all ASVs in this work, 953 ASVs were shared by three groups. In addition, 2,743 ASVs were detected in WCP group, while no was detected in control group, and 2,247 ASVs were detected in UCP group. However, different groups had their own separate sets of ASVs: the UCP group was 1930, the WCP group was 2,426, and the control group was 1,677. Simultaneously, there were 3,314, 3,512 and 4,068 ASVs each in control group, UCP group and WCP group. This implies that the species richness of the rat gut microbiota was enhanced by Cistanche polysaccharides.

#### 3.3.1 Examining the variations in the gut microbiome structure

When examining the microbial community’s composition in intestinal fecal samples, *α*-and *β*-diversity are frequently employed to assess the microbial community’s variation, complexity, and population makeup. When examining the microbial community’s composition in intestinal fecal samples, α- and β-diversity are frequently employed to assess the microbial community’s variation, complexity, and population makeup. With the use of the indices of Chao1, Observed species, Goods_coverage, Shannon, Simpson, and pielou-e, α-diversity primarily represents the richness and homogeneity. Chao1 and Observed_species provide an estimate of the number of species present in a community, which is known as abundance. Microbial coverage is expressed in Goods_coverage, which requires the coverage index to be equal to 1, that is, most of each base in the genome of the sample to be tested can be sequenced. The Pielou-e index represents evenness, while the Shannon and Simpson indices suggest diversity. Figures 5B-G shows that all five indices in the WCP group were considerably higher than those in the control group (*p* < 0.05), indicating that the administration of WCP improved community homogeneity and richness. The gut microbiota’s diversity was found to be improved by UCP, as evidenced by the greater Shannon and Simpson indices of the UCP group compared to the control group (*p* < 0.05). Conversely, when comparing the UCP group to the control group, there were no appreciable changes in any of the three indexes of Chao1, Observed species, and Pielou-e. This suggested that the UCP intervention had no discernible impact on the diversity of the gut microbiome. *β*-diversity is a measure of the variations in species between environmental communities. To perform β-diversity analysis, the distance matrix between any two samples in the environment must be calculated. Principal coordinate analysis (PCoA) (23) and cluster analysis (UPGMA) (24) are then used to identify sample differences. Figure 5H displays the findings of the PCoA analysis using the feature sequence, which is able to differentiate characteristics between various groups. The samples from the control group and the WCP group were separated by a considerable amount, suggesting that the two groups’ microbial structures and compositions were very different from one another. There was little variation in the microorganism structure and makeup of the UCP group and the control group, as evidenced by the close spacing between the samples in the two groups. The clustered tree grouping in the UPGMA diagram is represented by branches of different colors. The shorter the branch length, the more similar the two samples are, and the higher the similarity of the samples. Figures 5H,I illustrates how distinct the WCP group and the control group are from each other, and this visualization of the data could be used to graphically represent how the microbial evolution of the two groups is unique, suggesting that the WCP has the ability to regulate the intestinal flora of normal rats.

#### 3.3.2 Evaluating the gut microbiota’s composition

**Figure 6:**
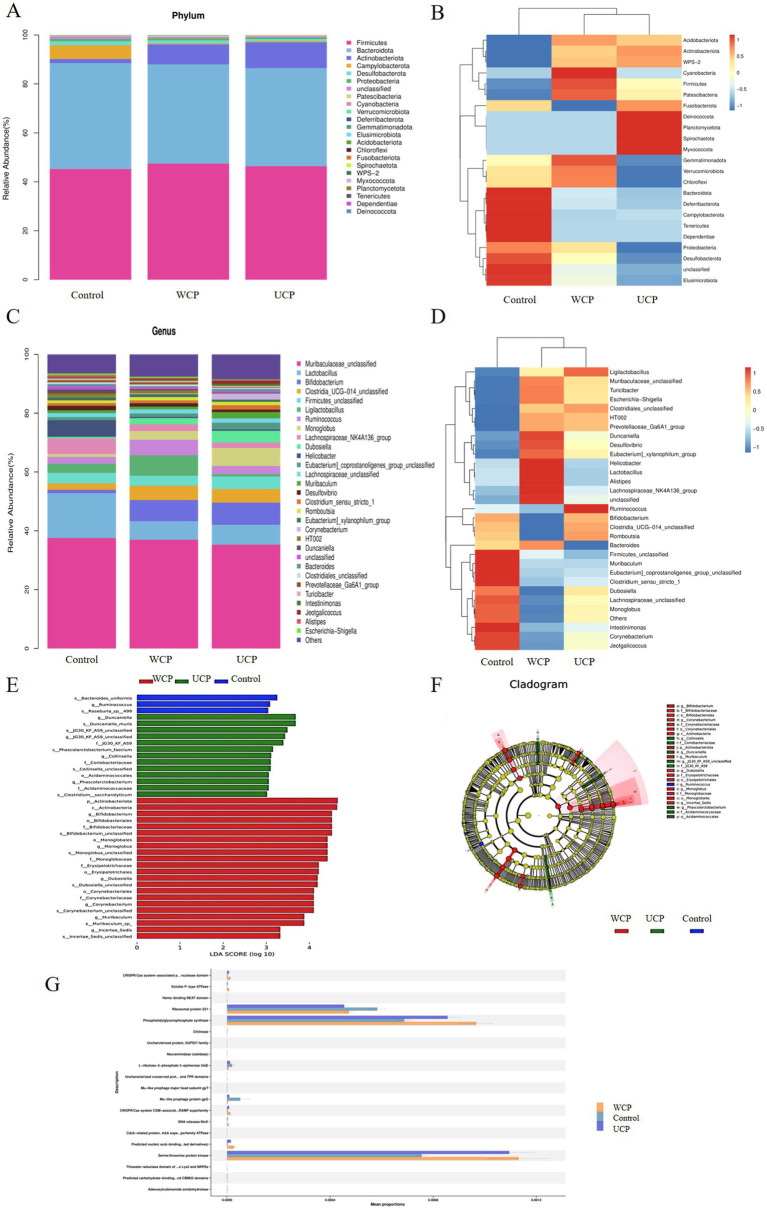
Relative abundance of the phylum-level microbial makeup **(A)**. A heatmap displaying the corresponding abundances of a given phylum, highlighting notable variations within each group **(B)**. The microbial composition’s proportions divided based on genus **(C)**. A heatmap showing the corresponding abundances of specific specified genera, with notable variations between each group **(D)**. Linear discriminant analysis (LDA) effect sizes were used to examine variations in microbial taxa among the three groups **(E)**. The taxa that belong in each group with the greatest differential abundance are indicated by LEfSe plots. **(F)**; PICRUSt2 functional annotation of pathway **(G)**. *n* = 6.

In the three groups, totals of 23 different phyla of gut microbiota were identified., and the phylum data with significant differences are shown in Supplementary Table S2. Among them *Firmicutes*, *Bacteroidota*, *Actinobacteriota*, *Campylobacterota*, *Desulfobacterota*, *Proteobacteria*, *Patescibacteria*, *Cyanobacteria*, *Verrucomicrobiota* are the main phylum. As Figures 6A,B illustrates, the distribution of these phylums is different among the three groups. Furthermore, the WCP group had a higher number of species detected at the phylum level than control group and the *Elusimicrobiota* phylum was newly added to the WCP. The abundance of *Firmicutes*, *Actinobacteriota*, *Patescibacteria*, *Cyanobacteria*, and *Verrucomicrobiota* increase during the same period. At the genus level, 31 genera of gut microbiota have been discovered, and the data with significant differences are shown in Supplementary Table S3. The groups WCP and UCP can increase *Bifidobacterium*, *Romboutsia* and decrease the *Lachnospiraceae_NK4A136_group* to varying degrees. *Ligilactobacillus* and *Duncaniella* were observed to be considerably higher in the WCP group according to the control group (Figures 6C–F).

#### 3.3.3 PICRUSt2 predicting function prediction

Microbiota and function were “mapped” with PICRUSt2 (25). The PICRUSt2 program was utilized to estimate the function based on the species annotation and OTU abundance data from the database. The samples were functionally annotated with KEGG COG, EC, KO, PFAM, TIGRFAM pathways, and STAMP was employed to do the comparison analysis. Figure 6G displays the prediction results with *p* < 0.05. With respect to abundance, the Cistanche polysaccharide-administered WCP and UCP groups were found to be more abundant than the Control group in the following pathways: TCA cycle I (prokaryotic), L-alanine biosynthesis superpathway, UDP-N-acetylglucosamine-derived O-antigen building blocks biosynthesis superpathway and peptidoglycan biosynthesis II (staphylococci). These pathways directly affect the metabolism of acteoside or lead to the production of metabolites into the bloodstream and exert pharmacological effects.

### 3.4 Effect of Cistanche polysaccharides on short-chain fatty acids (SCFAs) of rats

**Figure 7:**
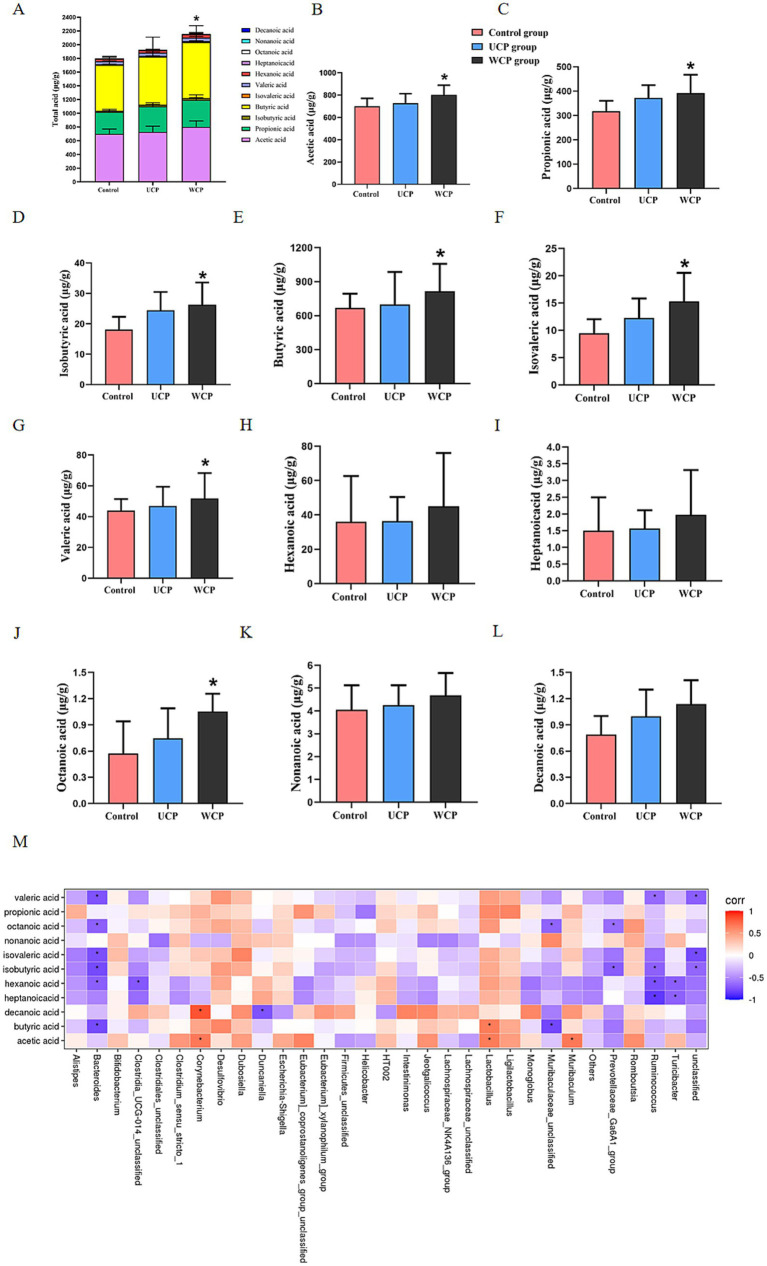
Cistanche polysaccharides administration increased the production of microbial SCFA metabolites. Measured by GC–MS in the stool of the Control, UPS, and WPS groups, **(A)** Total acid, **(B)** Acetic acid, **(C)** Propionic acid, **(D)** Isobutyric acid, **(E)** Butyric acid, **(F)** Isovaleric acid, **(G)** Valeric acid, **(H)** Hexanoic acid, **(I)** Heptanoicacid, **(J)** Octanoic acid, **(K)** Nonanoic acid, **(L)** Decanoic acid, and **(M)** Correlation heat map between fecal microbiota and fecal SCFAs content after Cistanche polysaccharides administration. ANOVA was used to establish significance, and values were reported as mean ± SD. *p < 0.05 in comparison to the control group.

Figure 7A illustrates that following 21 days of Cistanche polysaccharide administration, the levels of acetic acid, propionic acid, isobutyric acid, butyric acid, isovaleric acid, valeric acid, and octanoic acid were significantly elevated (*p* < 0.05) in the WCP group compared to the control group; the levels of acetic acid, propionic acid, isobutyric acid, butyric acid, isovaleric acid, valeric acid, and octanoic acid (*p* < 0.05) in the UCP group increased significantly than those in the control group (Figures 7B–L) but lower than those in the WCP group. The findings demonstrated that the intake of WCP and UCP both enhanced the quantity of SCFAs in the rat intestinal tract and could have improved the low bioavailability of oral acteoside in the gut. The effect of WCP is better than that of UCP.

In addition, the relationship between SCFA and gut microbiota was assessed. Figure 7M illustrates this relationship. SCFAs had a positive correlation (*p* < 0.05) with *Corynebacterium*, *Lactobacillus*, and *Muribaculum*, however a negative correlation (*p* < 0.05) with the gut microbes *Bacteroides*, *Ruminococcus*, *Turicibacter* and *Subdolilegum*.

## 4 Discussion

*Cistanche deserticola* is used as both food and medicine in China. Chemical analysis showed phenylethanoid glycoside and polysaccharides were the main active constituents. Acteoside, the main phenylethanoid in *Cistanche deserticola*, has anti-oxidation, sex hormones regulation (26), neuroprotective (27) and anti-inflammatory (28) biological activities, However, its druggability is limited due to the poor bioavailability, fast and extensive metabolism *in vivo*. Non-digested polysaccharides can serve as carriers or excipients in drug delivery systems, inhibiting the degradation of glycoside drugs in the intestine, improving drug absorption, increasing drug solubility, and enhancing drug bioavailability in vivo, achieving synergistic efficacy enhancement of drug (29). Cistanche polysaccharides induce core changes in the composition and diversity of intestinal microbiota and promote the absorption of echinacoside *in vivo* (30). Ginseng polysaccharides enhance ginsenoside Rb1 intestinal absorption by enhancing gut microbial metabolism and exposure to microbial metabolites (9). Soybean soluble polysaccharides modulate gut microbes and enhance the absorption of genistein in soybean in mice (31). These studies show that polysaccharides can promote the absorption of small molecule *in vivo* by regulating the diversity of the gut microbiota.

Chemical analysis of Cistanche polysaccharides showed that there was no significant change in the protein content of polysaccharides after rice-wine process. The total sugar content increased from 58.1 to 63.6%, and sugar yield increased from 22.82 to 25.02%. The increase of polysaccharide yield after being processed may be related to the hydrolysis of polysaccharides during the rice-wine steaming process (32, 33). The molecular weight for UCP and WCP was 22.868 kDa and 3.315 kDa separately. Results showed a significant change in the proportion of monosaccharide composition, with an increase in Glc and Ara content and a decrease in Gal and Xyl ratio in the WCP group.

**Figure 8:**
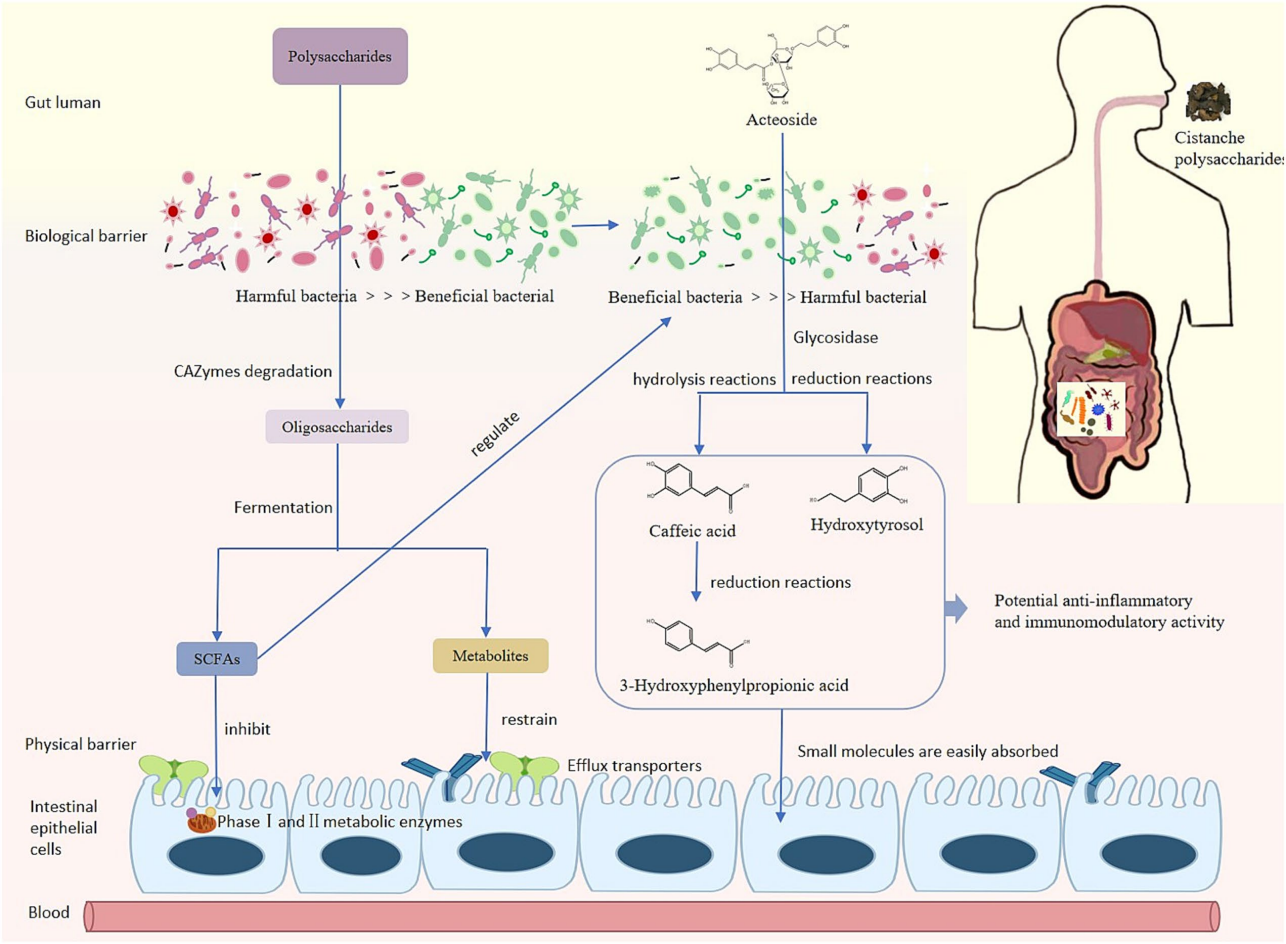
Metabolism process of phenylethanoid glycosides *in vivo*.

Pharmacokinetic studies have shown that C_max_ of acteoside increased by 1.19 times and 1.84 times, AUC(0-∞) increased by 1.16 times and 1.54 times after oral administration of UCP and WCP, respectively. According to literature reports, acteoside may generate caffeic acid, hydroxytyrosol, and 3-HPP through ester and glycosidic bond cleavage *in vivo* (34–37) (Figure 8) The C_max_ of caffeic acid in plasma increased by 1.45 times and 1.83 times, and the AUC(0-∞) increment rate can reach 142 and 214% times for hydroxytyrosol. Administration of WCP significantly increased the level of absorption of acteoside and its metabolites *in vivo*.

Cistanche polysaccharides induced core changes in abundance and diversity of gut microbiota. After 21 days of administration of WCP and UCP, there were differences in the composition and structure of the gut microbiota in rats. The abundance of *Firmicutes*, *Actinobacteriota*, *Patescibacteria* in WCP and UCP group was significantly increased, while the *Elusimicrobiota* phylum was newly added to the WCP. Gut *Firmicutes* has an important role in health, it is one of the main phylum in the human intestine (38), *Firmicutes* establishes a symbiotic relationship with the host through innate tolerance and resistance to control systemic immunity and avoid widespread inflammatory damage (39). The *Elusimicrobiota* phylum has been found to promote fatty acid and amino acid synthesis, and ultimately improve the growth (40). *Firmicutes* may have great potential in the treatment of metabolic and inflammatory diseases (41), suggesting that Cistanche polysaccharide administration promotes intestinal *Firmicutes* supplementation and thus exerts anti-inflammatory effects. Level of *Genus Bifidobacterium* and *Romboutsia* in WCP and UCP groups increased. *Bifidobacterium* as a type of intestinal degrading bacteria, maintains the balance of the intestinal environment and protects the host (42). *Romboutsia* can reduce the occurrence of inflammatory reactions (43). Reduced abundance of *Lachnospiraceae_NK4A136_group* in the WCP and UCP groups compared to the control group. High level of *Lachnospiraceae_NK4A136_group* can be observed in intestinal barrier damage rats induced by high-fat diet (HFD). Low levels of *Lachnospiraceae_NK4A136_group* can reduce the risk of intestinal pathogen colonization and inflammation (44). *Ligilactobacillus*, which is significantly increased in the WCP group, is a beneficial bacterium with anti-inflammatory effects both *in vivo* and *in vitro* (45). The WCP group significantly increased the abundance of *Duncaniella. Duncaniella* has been shown to protect the host from DSS-induced inflammatory colonic injury (46). These results show that the wine-processed *Cistanche deserticola* polysaccharides may regulate intestinal microorganisms and enhance anti-inflammatory ability.

The products of polysaccharides decomposition and metabolism by gut microbiota play an important role in various metabolic processes of the body. Intestinal flora decomposes polysaccharides by secreting enzymes (glycoside hydrolase, lyase, and esterase), produces short-chain fatty acids (SCFAs) including formic acid, acetic acid, propionic acid, and butyric acid (47). Cistanche polysaccharides improve intestinal microbiota disorders by influencing the structure and function of intestinal microbiota, inducing beneficial bacteria to produce a variety of metabolites such as short-chain fatty acids (SCFAs). Our experimental results showed that the content of acetic acid, butyric acid, propionic acid and isobutyric acid significantly increased in the WCP group.

Many factors affect the level of SCFAs in the feces, including the composition and the amount of gut microbiota, the dietary source (48). Accumulating studies shows that polysaccharides structure, including composition of monosaccharide residues, degree of polymerization, both can affect fermentation properties and SCFAs production (49). From our results, we illustrated that Cistanche polysaccharides modulate SCFAs production and polysaccharides of different processed products have different effect.

The correlation analysis showed that *Corynebacterium*, *Lactobacillus*, and *Muribaculum* were positively correlated with SCFAs, while *Bacteroides*, *Ruminococcus*, *Turicibacter* and *Subdolilegum* were negatively correlated. Butyric acid and propionic acid are produced by glycolysis of carbohydrates in the intestine through the action of *Firmicutes*; *Bacteroides* contribute to the formation of acetic acid and propionic acid in the intestine. Butyrate can regulate the intestinal immunity and inflammatory response of epithelial cells through inhibition of histone deacetylases (HDACs) and induction of anti-inflammatory cytokine expression (50), propionate exerts an immunomodulatory mechanism in acute myeloid leukemia by inducing ferroptosis (51). In this experiment, the content of butyrate and propionate was significantly increased in the WCP group. Cistanche polysaccharides, particularly wine-processed products, demonstrate prebiotic-like activity through SCFA-mediated immunomodulatory pathways that enhance acteoside bioavailability, optimize gut barrier integrity, and facilitate small molecule absorption via microbiota-mediated metabolic activation.

## 5 Conclusion

In this work, we developed a quick and easy-to-use UPLC-QqQ-MS technique and effectively used it to investigate the acteoside pharmacokinetics in rat plasma following oral administration of Cistanche polysaccharides. This study indicated that 21 days continuous administration of Cistanche polysaccharides can improve the composition and structure of intestinal microbiota, especially increased the level of *Ligilactobacillus* and *Duncaniella*, then promote the absorption of acteoside and its metabolites caffeic acid and hydroxytyrosol. The effect of WCP is superior to that of UCP. Comparative analysis revealed that both WCP and UCP exert comparable prebiotic-like effects on intestinal microbiota modulation, with WCP demonstrating superior capacity for short-chain fatty acid production and immune-enhancing bioactivities. They also promote higher C_max_ of acteoside and their metabolites, which is an effective way to enhance small molecule absorption and metabolism. In the further research, to clarify the mechanism by which polysaccharides from *Cistanche deserticola* increase the metabolism and absorption of small compounds *in vivo*, we will concentrate on the regulatory effects of the second phase metabolic enzymes and transporters of efflux in the inrastinal tract. In addition, we will examine gender bias and extended intervention time to assess the long-term effects of Cistanche polysaccharides on the microbiota and sustained improvement in bioavailability.

## Data Availability

The original contributions presented in the study are included in the article/Supplementary material, further inquiries can be directed to the corresponding authors.
