# Supplementary material for: Enhanced oral bioavailability of two Cistanche polysaccharides in acteoside: an in-depth analysis of intestinal flora, short-chain fatty acids, and pharmacokinetic regulation
Source: Front Nutr. 2025 May 2;12:1509734. doi: 10.3389/fnut.2025.1509734 (PMC12083017; doi:10.3389/fnut.2025.1509734)
Supplement: Supplementary file 1 [file Table_1.docx]

Supplementary Material

# Table S1 Data preprocessing statistics and quality control.

| Group | Sample | Raw Tags | Clean Tags | Valid% |
| --- | --- | --- | --- | --- |
| Control | 1 | 81985 | 67704 | 82.58 |
|  | 2 | 86191 | 78148 | 90.67 |
|  | 3 | 83909 | 70590 | 84.13 |
|  | 4 | 81862 | 68441 | 83.61 |
|  | 5 | 85218 | 75617 | 88.73 |
|  | 6 | 82679 | 74605 | 90.23 |
| UCP | 1 | 85710 | 76120 | 88.81 |
|  | 2 | 86705 | 79569 | 91.77 |
|  | 3 | 82229 | 73999 | 89.99 |
|  | 4 | 82414 | 75886 | 92.08 |
|  | 5 | 84107 | 72828 | 86.59 |
|  | 6 | 83457 | 75105 | 89.99 |
| WCP | 1 | 86802 | 76821 | 88.50 |
|  | 2 | 83745 | 72059 | 86.05 |
|  | 3 | 85867 | 75813 | 88.29 |
|  | 4 | 87505 | 76968 | 87.96 |
|  | 5 | 85479 | 73537 | 86.03 |
|  | 6 | 80907 | 69614 | 86.04 |

**Table S2** The relative abundance of dominant phyla at phylum level.

| Kinds | Control group | WCP group | UCP group |
| --- | --- | --- | --- |
| *Firmicutes* | 35.22±3.52 | 47.46±2.25* | 46.37±3.15* |
| *Actinobacteriota* | 1.68±0.20 | 8.19±0.59* | 10.56±0.73* |
| *Patescibacteria* | 0.26±0.02 | 0.36±0.01* | 0.32±0.02* |
| *Cyanobacteria* | 0.14±0.01 | 0.18±0.03* | 0.14±0.02 |
| *Verrucomicrobiota* | 0.14±0.03 | 0.19±0.08* | 0.06±0.02 |

Data are expressed as mean ± SD (n=6). **P* < 0.05 again Control group

# Table S3 The relative abundance of dominant taxa at genus level.

| Kinds | Control group | WCP group | UCP group |
| --- | --- | --- | --- |
| *Bifidobacterium* | 1.17±0.42 | 7.20±0.39* | 7.53±0.42* |
| *Ligilactobacillus* | 3.15±0.21 | 6.97±0.34* | 0.82±0.04 |
| *Lachnospiraceae_NK4A136_group* | 5.32±0.88 | 2.25±0.42* | 1.86±0.39* |
| *Romboutsia* | 0.92±0.03 | 1.12±0.04* | 1.09±0.01* |
| *Duncaniella* | 0.22±0.07 | 1.36±0.09* | 0.47±0.03* |

Data are expressed as mean±SD (n=6). **P* < 0.05 again Control group

# Table S4 The Linear relationship table of short chain fatty acids

| No. | Component | Retention time (min) | Linear equation | Correlation coefficient (R2) | Linear range (mg/L) |
| --- | --- | --- | --- | --- | --- |
| 1 | Acetic acid | 5.0600 | y = 0.0208x + 0.0034 | 0.9999 | 1~100 |
| 2 | Propionic acid | 5.9600 | y = 0.0097x + 0.0006 | 0.9998 | 1~50 |
| 3 | Isobutyric acid | 6.2350 | y = 0.0397x + 0.0013 | 0.9999 | 0.1~5 |
| 4 | Butyric acid | 6.9200 | y = 0.0353x + 0.0073 | 1.0000 | 1~100 |
| 5 | Isovaleric acid | 7.3450 | y = 0.0402x + 0.0012 | 0.9999 | 0.2~5 |
| 6 | Valeric acid | 8.1000 | y = 0.0435x + 0.0007 | 0.9999 | 0.2~10 |
| 7 | Hexanoic acid | 9.2200 | y = 0.0388x + 0.0021 | 0.9999 | 0.2~10 |
| 8 | Heptanoicacid | 10.2950 | y = 0.0373x + 0.0003 | 1.0000 | 0.02~1 |
| 9 | Octanoic acid | 11.3300 | y = 0.0338x + 0.0004 | 1.0000 | 0.02~1 |
| 10 | Nonanoic acid | 12.3150 | y = 0.0298x + 0.0004 | 0.9994 | 0.02~1 |
| 11 | Decanoic acid | 13.2650 | y = 0.0296x + 1E-05 | 0.9999 | 0.02~1 |

# Table S5 Recovery of short-chain fatty acids（n=6）

| Compound | Initial concentration | QC1(mg/L) | Recovery rate(%) | QC2(mg/L) | Recovery rate(%) | QC3(mg/L) | Recovery rate(%) | Average recovery | RSD (%) |
| --- | --- | --- | --- | --- | --- | --- | --- | --- | --- |
| Acetic acid | 1mg/L | 0.83 | 83 | 0.850660998 | 85.07 | 0.83 | 83 | 83.68869992 | 0.014253576 |
| Propionic acid | 1mg/L | 1.04 | 104 | 1.03041659 | 103.04 | 1.03 | 103 | 103.3472197 | 0.005473857 |
| Isobutyric acid | 1mg/L | 0.94 | 94 | 0.928054094 | 92.81 | 0.95 | 95 | 93.93513648 | 0.011696711 |
| Butyric acid | 1mg/L | 0.85 | 85 | 0.844657446 | 84.47 | 0.83 | 83 | 84.15524819 | 0.012304902 |
| Isovaleric acid | 1mg/L | 0.97 | 97 | 0.955477288 | 95.55 | 0.99 | 99 | 97.18257627 | 0.017836142 |
| Valeric acid | 1mg/L | 0.98 | 98 | 0.957142015 | 95.71 | 0.98 | 98 | 97.23806717 | 0.013571911 |
| Hexanoic acid | 1mg/L | 0.98 | 98 | 0.956562196 | 95.66 | 0.99 | 99 | 97.55207319 | 0.01759371 |
| Heptanoicacid | 1mg/L | 0.97 | 97 | 0.939989522 | 94.00 | 0.98 | 98 | 96.33298407 | 0.021615164 |
| Octanoic acid | 1mg/L | 0.92 | 92 | 0.901880478 | 90.19 | 0.91 | 91 | 91.06268261 | 0.009966772 |
| Nonanoic acid | 1mg/L | 1.16 | 116 | 1.088901594 | 108.89 | 1.12 | 112 | 112.2967198 | 0.031739089 |
| Decanoic acid | 1mg/L | 0.89 | 89 | 0.854318936 | 85.43 | 0.89 | 89 | 87.8106312 | 0.023460111 |
